# Prevalence of depression and anxiety in systemic lupus erythematosus: a systematic review and meta-analysis

**DOI:** 10.1186/s12888-017-1234-1

**Published:** 2017-02-14

**Authors:** Lijuan Zhang, Ting Fu, Rulan Yin, Qiuxiang Zhang, Biyu Shen

**Affiliations:** 1 Department of Nursing, The Second Affiliated Hospital of Nantong University, 6th Haierxiang Road, 226001 Nantong, People’s Republic of China; 2 School of Nursing, Nantong University, Nantong, People’s Republic of China

**Keywords:** Depression, Anxiety, Meta-analysis, Systematic review

## Abstract

**Background:**

Systemic lupus erythematosus (SLE) patients are at high risk for depression and anxiety. However, the estimated prevalence of these disorders varies substantially between studies. This systematic review aimed to establish pooled prevalence levels of depression and anxiety among adult SLE patients.

**Methods:**

We systematically reviewed databases including PubMed, Embase, PsycINFO, and the Cochrane database library from their inception to August 2016. Studies presenting data on depression and/or anxiety in adult SLE patients and having a sample size of at least 60 patients were included. A random-effect meta-analysis was conducted on all eligible data.

**Results:**

A total of 59 identified studies matched the inclusion criteria, reporting on a total of 10828 adult SLE patients. Thirty five and thirteen methods of defining depression and anxiety were reported, respectively. Meta-analyses revealed that the prevalence of major depression and anxiety were 24% (95% CI, 16%-31%, I^2^ = 95.2%) and 37% (95% CI, 12%–63%, I^2^ = 98.3%) according to clinical interviews. Prevalence estimates of depression were 30% (95% CI, 22%–38%, I^2^ = 91.6%) for the Hospital Anxiety and Depression Scale with thresholds of 8 and 39% (95% CI, 29%–49%, I^2^ = 88.2%) for the 21-Item Beck Depression Inventory with thresholds of 14, respectively. The main influence on depression prevalence was the publication years of the studies. In addition, the corresponding pooled prevalence was 40% (95% CI, 30%–49%, I^2^ = 93.0%) for anxiety according to the Hospital Anxiety and Depression Scale with a cutoff of 8 or more.

**Conclusions:**

The prevalence of depression and anxiety was high in adult SLE patients. It indicated that rheumatologists should screen for depression and anxiety in their patients, and referred them to mental health providers in order to identify effective strategies for preventing and treating depression and anxiety among adult SLE patients.

**Trial registration:**

Current Meta-analysis PROSPERO Registration Number: CRD 42016044125. Registered 4 August 2016.

**Electronic supplementary material:**

The online version of this article (doi:10.1186/s12888-017-1234-1) contains supplementary material, which is available to authorized users.

## Background

Systemic lupus erythematosus (SLE) is a multisystem, autoimmune, connective-tissue disorder with frequent psychological comorbidities, of which depression and anxiety are two common manifestations [1, 2]. It has been reported that there were 2 times higher prevalence of depression in SLE patients compared to the general population [3]. In addition, previous study has reported that the anxiety disorders were twice as prevalent among SLE patients as compared to the controls [4]. Depression and anxiety often have profound impacts on SLE patients’ health and well-being including increased incidence of cardiovascular diseases [5], myocardial infarction [6], suicidal ideation [7], physical disability [8], decreased quality of life [9, 10], and a higher risk of premature mortality [11]. Therefore, depression and anxiety may be useful targets for interventions aimed at improving subjective health and quality of life in individuals with SLE. However, current epidemiological evidence found that the prevalence of depression and/or anxiety in SLE patients ranged widely from 2% to 91.7% in different studies [12, 13]. This vast inter-study difference was previously attributed to multiple factors, including study quality, unclear definition of depression or anxiety, diverse screening strategies used across studies [14]. Reliable estimates of depression and anxiety prevalence are important for informing efforts to prevent, treat, and identify causes of depression and anxiety among SLE patients. Recent meta-analyses have estimated the overall prevalence of depression and/or anxiety in rheumatoid arthritis and osteoarthritis patients [14, 15]. There has only been one previous systematic review of psychiatric symptoms in SLE [16]; however, no systematic review was conducted to quantify the prevalence of depression and anxiety in SLE using meta-analysis techniques. Our goal was to address this limitation. The objectives of this systematic review were (i) to establish pooled prevalence levels of depression and anxiety among adult SLE patients; (ii) to provide a summary of the methods used to define depression and anxiety in SLE; and (iii) to explore the impacts of study characteristics on prevalence estimates.

## Methods

This systematic review was conducted within the Reporting Items for Systematic reviews and Meta-Analyses (PRISMA) statement [17] and followed a predetermined registered protocol (PROSPERO: CRD42016044125).

### Search strategy

A systematic review of published literature in scientific journals that reported on the prevalence of depression and/or anxiety among SLE patients was conducted by two independent reviewers using the following databases from their inception to August 2016: PubMed, Embase, PsycINFO, and the Cochrane database library. The computer-based searches combined terms related to SLE patients and study design with those related to depression or anxiety (see Additional file 1). We conducted citation chasing search strategy with all reference lists of included articles and relevant review papers were considered to identify potentially omitted articles. Finally, we corresponded with the authors for further information if we encountered articles just provided the mean and standard deviation of the depression and/or anxiety assessment scale.

### Inclusion and exclusion criteria

Studies were included if they met the following criteria: (i) cross-sectional design, baseline cross-sectional data from a longitudinal study or baseline cross-sectional data from a trial, before group allocation; (ii) used validated methods (clinical interviews or self-report instruments) to assess depression or anxiety; and (iii) the sample size was no less than 60.

Case reports, review articles, animal studies, studies investigating neuropsychiatric syndromes, studies in languages other than English and papers not dealing with SLE patients were excluded. For this meta-analysis, studies using pediatrics sample or screening tools without stating the cut-off thresholds used to detect depression or anxiety were also excluded. Table 2 and Table 3 presented a full list of the eligible methods of detecting depression and anxiety, alongside the numbers of articles utilizing each method and the number of participants assessed.

### Data extraction and quality assessment

Two researchers read the relative studies independently by the titles and abstracts to exclude the references which did not met the inclusion criteria. Then, they read full texts in the remaining studies as mentioned above, and determined whether these references included were final studies or not. When multiple publications spanned the years of longitudinal studies, baseline prevalence levels were reported. The following information was independently extracted from each article by other two trained investigators using a standardized form: year, country, mean disease duration, percentage of female participants, sample size, average age of participants, criteria for detection of depression and anxiety, and reported prevalence of depression and/or anxiety. If we encountered multiple publications from the same cohort, we used the data from the most recent or the paper reporting data from the largest number of participants. The methodological quality of each study included in the present meta-analysis was assessed using a modified version of the Newcastle-Ottawa Scale [18]. Studies were judged to be at low risk of bias (≥3 points) or high risk of bias (<3 points). Any disagreements in data extraction and quality assessment were resolved through discussion between the two reviewers or adjudication with a third reviewer.

### Outcome measures

The outcomes were major/minor depression and affective/dysthymic/adjustment/anxiety disorder diagnosed with a structured clinical assessment [e.g., Diagnostic and Statistical Manual of Mental Disorders (DSM)-IV or International Classification of Diseases (ICD)-10] or depression and/or anxiety assessed with validated assessment tools [e.g., the Hospital Anxiety and Depression Scale (HADS), the Centre for Epidemiologic Studies Depression Scale (CES-D)] (see Additional file 2).

### Statistical analyses

Because random-effects models tended to provide wider confidence intervals (CI) and were preferable in the presence of between-study heterogeneity, we used a random-effects meta-analysis to pool studies reporting the prevalence of depression and/or anxiety in SLE patients [19]. Between-study heterogeneity was assessed by the I^2^ with thresholds of ≥25%, ≥50% and ≥75% indicating low, moderate and high heterogeneity, respectively [20]. The influence of individual study on the overall prevalence estimate was explored by serially excluding each study in sensitivity analyses. Wherever possible, subgroup analyses were planned by overall study quality, sample size, country of origin and publication year, if there was more than one study in the subgroup. Pearson’s and Spearman’s correlation analyses were used to assess the association between variables and prevalence of depression and anxiety in people with SLE. Funnel plots and Egger’s test were combined to explore the potential publication bias in this meta-analysis [21, 22]. Statistical analyses were performed with STATA version 12.0. Statistical tests were 2-sided and used a significance threshold of *P* < 0.05.

## Results

### Search results

**Fig. 1:**
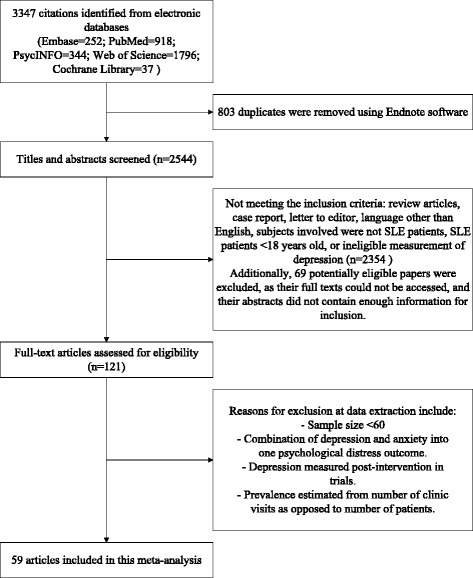
Search results and study selection

Fig. 1 provided the details of the study selection process. The initial search identified a total of 3347 potentially relevant articles. After removal of duplicates, titles and then abstracts were screened for potential eligibility. From this, 121 were considered in the full-text review, of which 59 articles met the inclusion criteria, and a full reference list was presented in Additional file 3. Inter-rater reliability of reviewers regarding study relevancy was high (Kappa = 0.87).

### Study characteristics

A summary of the included study characteristics was shown in Table 1. A total of 59 identified studies matched the inclusion criteria, reporting on a total of 10828 adult SLE patients. Twenty took place in North America, 18 in Asia, 12 in Europe, 6 in South America, 1 in Oceania, and 1 in Africa. The median of mean ages was 39 years (range, 30.0-50.1), and the median percentage of females represented in the sample was 93% (range, 75%–100%). In addition, the median number of participants per study was 100 (range, 60–1827), and the median of mean disease duration was 9 years (range, 0.22–16.3). Depression was defined in 35 different ways (Table 2). Seventeen studies assessed for depression using the 21 Item-Beck Depression Inventory (BDI), with sixteen different thresholds were presented in the articles. Thirteen articles used the CES-D; six different cut-off points were presented, and the most commonly used being 16. Twelve used the HADS with a cutoff of 8 or more, and 6 used other screening tools. Ten studies assessed for major depression using diagnostic criteria (DSM or ICD). The most commonly used screening questionnaire to assess anxiety was the HADS, with 10 studies using this screening tool with thresholds of 8. The methods employed to assess depression and anxiety and the frequency of their use were presented in Table 2 and Table 3. When evaluated by Newcastle-Ottawa quality assessment criteria, out of 5 possible points, 2 studies received 5 points, 7 received 4 points, 13 received 3 points, 36 received 2 points, and 1 received 1 point. The details of the assessment of individual studies were shown in Additional file 4.

**Table 1:** Overview of prevalence studies of mood in SLE patients (N ≥ 60)

Study ID	Country	Disease duration, mean ± SD/median (range)	Women, %	Sample size	Age, mean ± SD/median (range), years	Criteria for detection of anxiety (cutoff)	Anxiety prevalence, %	Criteria for detection of depression (cutoff)	Depression prevalence, %	NOS
Abdul-Sattar 2015	Egypt	10.0 ± 4.6 years	95%	80	30.9 ± 11.7			CES-D (>16.7)	43.75	2
Appenzeller 2009	Brazil	64.5 ± 48.5 months	94.6%	167	32.1 ± 11.0			21 Item-BDI (≥10)	20.9	2
Bachen 2009	USA	15.4 ± 9.7 years	100%	326	47.9 ± 11.3	DSM-IV	64	DSM-IV	Major depressive disorder: 42.4, dysthymic disorder: 2.9	5
Bogdanovic 2015	Serbia	6.8 ± 2.9 years	100%	60	43.4 ± 12.8			21 Item-BDI (≥16/≥20/≥30)	91.7/70/3.3	2
Calderon 2014	Chile	Median: 32.0 (0–243.0) months	100%	82	Median: 36.0 (17.0–64.0)			HADS (≥8)	37	2
Cho 2014	South Korea	NS	90.1%	201	41.3 ± 13.2			CES-D (≥16)	39.3	3
Chin 1993	Malaysia	4.1 ± 3.5 years	95%	79	31.1 ± 9.1	ICD-9 and DSM-III	7.6	ICD-9 and DSM-III	Major depressive disorder: 6.3, dysthymic disorder: 32.9	2
Da Costa 2005	Canada	13.8 ± 10.1 years	100%	100	45.4 ± 14.0			CES-D (≥16)	31	3
Doria 2004	Italy	9.9 ± 6.3 years	87.3%	126	38.9 ± 11.9	HAS (≥6/≥15)	74.6/27	HDS (≥8/≥16)	40.5/2.4	2
Duvdevany 2011	Israel	11.4 ± 9.1 years	88%	100	37.0 ± 11.8	HADS (≥8)	20	HADS (≥8)	37	4
García-Carrasco 2011	Mexico	106.5 ± 85.5 months	100%	106	40.5 ± 12.0			CES-D (≥16)	38.8	2
García-Carrasco 2013	Mexico	10.5 ± 7.4 years	100%	105	43.6 ± 11.3			CES-D (≥16)	33	2
Greco 2009	USA	16.3 ± 7.0 years	100%	161	50.1 ± 10.0			CES-D (≥16)	27	2
Hanly 2015	Canada	5.6 ± 4.8 years	88.9%	1827	35.1 ± 13.3			DSM-IV	12.7	4
Harrison 2006	USA	15.3 ± 3.2 years	100%	93	43.3 ± 13.7			CES-D (>27)	16.1	2
Huang 2007	China	7.5 ± 6.9 years	91.5%	129	37.4 ± 10.7	HADS (≥8)	32	HADS (≥8)	20	2
Iverson 2002	Canada	NS	NS	103	NS			21 Item-BDI (≥17)	39.8	1
Jarpa 2011	Chile	Median: 5.0 (0.1–40.0) years	90.8%	87	Median: 39.0 (16.0–27.0)	DSM-IV	18.1	DSM-IV	Major depressive disorder: 21.7, dysthymic disorder: 4.8	2
Julian 2011	USA	15.8 ± 9.3 years	93%	150	48.8 ± 12.3			ICD-10 and DSM-IV	Major depressive disorder: 17, dysthymic disorder: 4, minor depression: 6	3
Jung 2015	Korea	6.8 ± 4.4 years	93%	100	40.6 ± 10.3			21 Item-BDI (≥21)	13	2
Katz 2011	USA	13.6 ± 8.5 years	100%	716	48.1 ± 12.6			CES-D (≥24)	25	3
Karol 2013	USA	NS	93%	127	38.1 ± 12.3			21 Item-BDI (≥18)	41.7	2
Karimifar 2013	Iran	4.1 ± 0.5 years	80%	100	34.8 ± 10.9			21 Item-BDI (≥14)	60	2
Kheirandish 2015	Iran	9.0 ± 7.7 years	92.2%	166	33.1 ± 11.1	Cattell questionnaire (≥21)	84.9	21 Item-BDI (≥5/≥30)	64.5/9	2
Kotsis 2014	Greece	13.2 ± 9.1 years	84%	75	44.1 ± 13.3			PHQ-9 (≥10)	29.3	2
Kim 2015	USA	12.0 ± 8.0 years	93%	89	39.0 ± 15.0			CES-D (≥16)	63	3
Lapteva 2006	USA	13.8 ± 10.2 years	75%	60	41.0 ± 13.0			DSM-IV	Major depressive disorder: 16.6	2
Lisitsyna 2014	NS	134.9 ± 8.8 months	85.6%	180	34.6 ± 0.93			ICD-10	Major depressive disorder: 24.4, dysthymic disorder: 25.6, adjustment disorders: 18.9	2
Mak 2011	Singapore	54.9 ± 70.7 months	88%	60	40.5 ± 12.9	HADS (≥8)	38	HADS (≥8)	22	2
Maneeton 2013	Thailand	6.1 ± 4.8 years	98%	62	31.8 ± 9.0	HAS (≥14)	37.1	HDS (≥11)	45.2	2
Mirbagher 2016	Iran	8.3 ± 3.8 years	100%	77	36.5 ± 10.1	HADS (≥8)	71.4	HADS (≥8)	46.1	3
Monaghan 2007	Australia	10.2 ± 8.7 years	97%	60	44.4 ± 12.2	HADS (≥8)	44	HADS (≥8)	36	3
Montero-Lo’pez 2016	Spain	0.2 ± 0.7 years	100%	97	38.6 ± 9.3	SCL-90-R	4.1	SCL-90-R	5.2	2
Nery 2008	Brazil	9.8 ± 6.5 years	100%	71	34.8 ± 10.1	SCID for DSM-IV	46.5	SCID for DSM-IV	Major depressive disorder: 40.8	2
Neville 2014	Canada	10.2 ± 9.5 years	92.4%	612	46.8 ± 16.7			PHQ-2 (≥3)	28.1	4
Palagini 2014	Italy	15.0 ± 8.0 years	100%	81	43.6 ± 11.2	SAS (>44)	17.3	21 Item-BDI (≥11)	34.6	3
Panopalis 2010	USA	13.8 ± 8.9 years	91%	807	47.6 ± 13.1			CES-D (≥16)	38.5	5
Pettersson 2015	Sweden	Median: 12.0 years	92%	305	Median: 48	HADS (≥8)	34	HADS (≥8)	51	4
Postal 2016	Brazil	Median: 9.0 (0–33.0) years	96.7%	153	Median: 30.0 (10.0–62.0)	21 Item-BAI (≥8/≥16/≥26)	60.7/43.1/18.3	21 Item-BDI (≥14/≥20/≥29)	45.7/30.7/18.9	2
Radhakrishan 2011	India	NS	100%	100	18-60	SCID for DSM-IV	51	SCID for DSM-IV	Major depressive disorder: 46, adjustment disorder: 21, dysthymic disorder: 9	2
Roebuck-Spencer 2006	USA	13.8 ± 10.2 years	80%	60	41.3 ± 12.8			21 Item-BDI (≥14)	20	2
Segal 2012	USA	12.0 ± 2.3 years	93%	71	41.7 ± 1.5			CES-D (≥16)	39	2
Sehlo 2013	Saudi Arabia	6.9 ± 4.2 years	100%	80	34.8 ± 11.2			SCID for DSM-IV	Major depressive disorder: 11.25	2
Sfikakis 1998	Greece	7.8 ± 6.4 years	91.5%	71	37.0 ± 13.0	HAS (>17)	23.9	HDS (>17)	19.7	2
Shakeri 2015	Iran	NS	92.5%	160	30.1 ± 6.2	21 Item-BAI (≥8/≥16/≥26)	81.2/51.9/18.1	21 Item-BDI (≥21/≥31/>40)	69.3/38.7/20.6	2
Shen 2015	China	NS	91.2%	156	32.9 ± 10.2	Zung SAS (≥50)	20.51	Zung SDS (≥53)	33.33	3
Skare 2014	Brazil	8.2 ± 6.9 years	93%	100	39.2 ± 12.5			21 Item-BDI (≥19/≥ 30)	21/2	2
Shorta1l 1995	England	11.0 ± 7.1 years	95%	80	41.0 ± 11.2	HADS (≥8)	39	HADS (≥8)	26	2
Stoll 2001	Switzerland	11.4 ± 9.0 years	90%	60	44.5 ± 15.4			HADS (≥8)	16	3
Tam 2008	China	9.7 years	95.9%	291	42.0 ± 12.0	HADS (≥8)	22	HADS (≥8)	18.2	3
Tay 2015	Singapore	72.3 ± 81.1 months	86.4%	110	38.7 ± 12.6	HADS (≥8)	40.9	HADS (≥8)	15.5	2
Tench 2000	England	Median: 36.0 (12.0–79.5) months	100%	120	Median: 38.0 (32.0–45.0)	HADS (≥8)	60	HADS (≥8)	37	2
Tjensvoll 2010	Norway	12.3 ± 8.6 years	87%	63	43.4 ± 13.3			21 Item-BDI (≥13)	23.8	2
Utset 2014	USA	Median: 9 years	95%	344	>18			CES-D (>10)	54.5	4
van Exel 2013	Netherlands	7.8 ± 7.0 years	88.2%	102	44.4 ± 12.5			21 Item-BDI (≥14)	27	3
Vina 2015	USA	143.2 ± 117.8 months	93%	343	44.4 ± 12.9			CES-D (≥17)	47.2	4
Weder-Cisneros 2004	USA	Mean: 97.0 (6–348) months	91.4%	81	31.2 ± 9.7			21 Item-BDI (≥14)	40.7	3
Xie 2012	China	Median: 1.3 years	93.7%	285	34.0 ± 13.0			21 Item-BDI (≥5/14/≥21)	59.3/40.7/19.3	4
Zakeri 2012	Iran	NS	90.5%	71	>18			21 Item-BDI (≥16/≥32)	60/9.4	2

*NS* not stated, *CES-D* Centre for Epidemiological Studies Depression Scale, *BDI* Beck Depression Inventory, *BAI* Beck Anxiety Inventory, *DSM-III/IV* Diagnostic and Statistical Manual of Mental Disorders, Third/Fourth Edition, *HADS* Hospital Anxiety and Depression Scale, *ICD* International Classification of Diseases, *HAS* the Hamilton Anxiety Scale, *HDS* the Hamilton Depression Scale, *PHQ* Patient Health Questionnaire, *SCID* Structured Clinical Interview for Diagnostic and Statistical Manual, *SCL-90-R* Symptoms Checklist-90-Revised, *Zung SAS* Zung Self-rating Anxiety Scale, *Zung SDS* Zung Self-rating Depression Scale

**Table 2:** Methods of detecting depression and summary of prevalence and heterogeneity findings

Tool	Definition/cutoff	No. of studies	No. of participants	Prevalence, % (95% CI)	Heterogeneity I^2^, %
DSM and/or ICD					
Major depressive disorder		10	2960	24 (16, 31)	95.2
Dysthymic disorder		6	922	12 (5, 18)	93.4
Adjustment disorder		2	280	20 (15, 24)	0.0
Minor depression		1	150	6 (2, 10)	-
HADS	≥8	12	1474	30 (22, 38)	91.6
CES-D	>10	1	344	55 (49, 60)	-
	≥16	8	1640	38 (32, 44)	81.3
	>16.7	1	80	44 (33, 55)	-
	≥17	1	343	47 (42, 52)	-
	≥24	1	716	25 (22, 28)	-
	>27	1	93	16 (9, 24)	-
21 Item-BDI	≥5	2	451	61 (56, 66)	17.7
	≥10	1	167	21 (15, 27)	-
	≥11	1	81	35 (24, 45)	-
	≥13	1	63	24 (13, 34)	-
	≥14	6	781	39 (29, 49)	88.2
	≥16	2	131	76 (45, 107)	95.4
	≥17	1	103	40 (30, 49)	-
	≥18	1	127	42 (33, 50)	-
	≥19	1	100	21 (13, 29)	-
	≥20	2	213	50 (12, 89)	96.8
	≥21	3	545	34 (2, 65)	98.8
	≥29	1	153	19 (13, 25)	-
	≥30	3	326	5 (0, 9)	72.1
	≥31	1	160	39 (31, 46)	-
	≥32	1	71	9 (3, 16)	-
	>40	1	160	21 (14, 27)	-
HDS	≥8	1	126	41 (32, 49)	-
	≥11	1	62	45 (33, 58)	-
	≥16	1	126	2 (0, 5)	-
	>17	1	71	20 (10, 29)	-
PHQ-9	≥10	1	75	29 (19, 40)	-
PHQ-2	≥3	1	612	28 (25, 23)	-
SCL-90-R		1	97	5 (1, 10)	-
Zung SDS	≥53	1	156	33 (26, 41)	-

*DSM* Diagnostic and Statistical Manual of Mental Disorders, *ICD* International Classification of Diseases, *HADS* Hospital Anxiety and Depression Scale, *CES-D* Centre for Epidemiological Studies Depression Scale, *BDI* Beck Depression Inventory, HDS Hamilton Depression Scale, *PHQ* Patient Health Questionnaire, *SCL-90-R* Symptoms Checklist-90-Revised, *Zung SDS* Zung Self-rating Depression Scale

**Table 3:** Methods of detecting anxiety and summary of prevalence and heterogeneity findings

Tool	Definition/cutoff	No. of studies	No. of participants	Prevalence, % (95% CI)	Heterogeneity I^2^, %
DSM and/or ICD for anxiety disorder		5	663	37 (12, 63)	98.3
HADS	≥8	10	1332	40 (30, 49)	93.0
21 Item-BAI	≥8	2	313	71 (51, 91)	94
	≥16	2	313	48 (39, 56)	59.2
	≥26	2	313	18 (14, 22)	0
HAS	≥6	1	126	75 (67, 82)	-
	≥14	1	62	37 (25, 49)	-
	≥15	1	126	27 (19, 35)	-
	>17	1	71	24 (14, 34)	-
Cattell questionnaire	≥21	1	166	85 (79, 90)	-
SCL-90-R		1	97	4 (0, 8)	-
Zung SAS	>44	1	81	17 (9, 26)	-
	≥50	1	156	21 (14, 27)	-

*DSM* Diagnostic and Statistical Manual of Mental Disorders, *ICD* International Classification of Diseases, *HADS* Hospital Anxiety and Depression Scale, *BAI* Beck Anxiety Inventory, *HAS* Hamilton Anxiety Scale, *SCL-90-R* Symptoms Checklist-90-Revised, *Zung SAS* Zung Self-rating Anxiety Scale

### Prevalence of depression among SLE patients

**Fig. 2:**
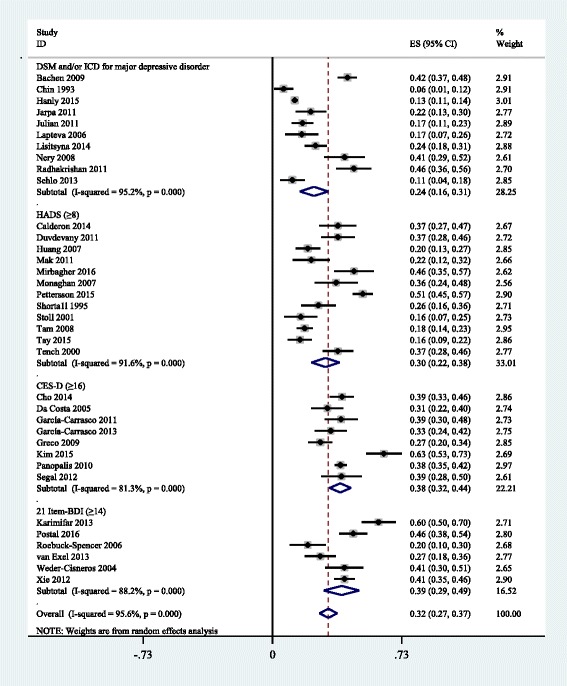
Prevalence of depressive disorder in SLE

Prevalence estimates of depression ranged from 2% to 91.7% in individual studies (Table 1). Table 2 indicated the summary of meta-analyses and heterogeneity assessments. Meta-analyses revealed the prevalence of major depressive disorder to be 24% (95% CI, 16%–31%) according to the DSM and/or ICD diagnostic criteria, with high heterogeneity (I^2^ = 95.2%). Prevalence estimates of depression were 30% (95% CI, 22%–38%, I^2^ = 91.6%) for the HADS with thresholds of 8 and 38% (95% CI, 32%–44%, I^2^ = 81.3%) for the CES-D with thresholds of 16, respectively. Prevalence of depression according to the 21 Item-BDI with a cutoff of 14 or more was 39% (95% CI, 29%–49%), with high heterogeneity (I^2^ = 88.2%) (Fig. 2).

### Prevalence of anxiety among SLE patients

**Fig. 3:**
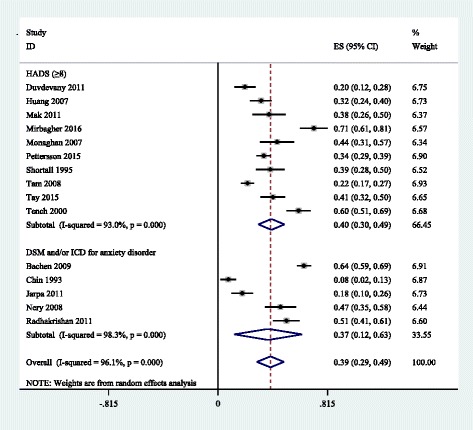
Prevalence of anxiety in SLE

Prevalence of anxiety alone ranged between 4% and 85% in individual studies (Table 1). Table 3 presented the summary of meta-analyses and heterogeneity assessments. Meta-analyses pooled the prevalence of anxiety to be 40% (95% CI, 30%–49%, I^2^ = 93.0%) and 37% (95% CI, 12%–63%, I^2^ = 98.3%) according to the HADS with thresholds of 8 and the DSM and/or ICD diagnostic criteria, respectively (Fig. 3).

### Sensitivity and subgroup analyses

Table 4 suggested depression and anxiety prevalence estimates according to each sensitivity and subgroup analysis, in comparison with the primary analysis. Sensitivity analyses revealed that the exclusion of studies with less sample representativeness tended to decrease dysthymic disorder prevalence estimates according to DSM and/or ICD. The removal of studies with less comparable respondent and non-respondent comparability tended to increase depression prevalence estimates according to the HADS with a cutoff of 8 or more. According to DSM and/or ICD, anxiety prevalence estimates had a trend to decrease by exclusion of studies only using female sample. The subgroup analyses were conducted according to sample size, overall quality, publication year, and country of origin. The results showed that studies with sample size <200 had higher anxiety estimates [43% (95% CI, 31%–55%) vs 28% (95% CI, 16%–40%)] according to the HADS with a cutoff of 8 or more. When evaluated by Newcastle-Ottawa criteria, studies with lower total overall quality scores yielded higher dysthymic disorder estimates [18% (95% CI, 6%–29%) vs 3% (95% CI, 2%–25%)] according to DSM and/or ICD. In contrast with clinical interviews (DSM and/or ICD), more recent publications tended to yield higher depression and anxiety prevalence estimates according to self-report instruments. The subgroup analyses for country of origin showed no clear patterns. There was no particular trend or pattern in any other sensitivity analyses or subgroup analyses.

**Table 4:** Impact of study characteristics on prevalence estimates for depression and anxiety in SLE: sensitivity and subgroup analyses

Depression definition (cutoff)								Anxiety definition (cutoff)	
	Major depressive disorder (DSM and/or ICD)	Dysthymic disorder (DSM and/or ICD)	HADS (≥8)	CES-D (≥16)	21 Item-BDI (≥14)	21 Item-BDI (≥21)	21 Item-BDI (≥30)	HADS (≥8)	Anxiety disorder (DSM and/or ICD)
Primary analysis	24 (16, 31) I^2^ = 95.2% 10 studies 2960 patients	12 (5, 18) I^2^ = 93.4% 6 studies 922 patients	30 (22, 38) I^2^ = 91.6% 12 studies 1474 patients	38 (32, 44) I^2^ = 81.3% 8 studies 1640 patients	39 (29, 49) I^2^ = 88.2% 6 studies 781 patients	34 (2, 65) I^2^ = 98.8% 3 studies 545 patients	5 (0, 9) I^2^ = 72.1% 3 studies 326 patients	40 (30, 49) I^2^ = 93.0% 10 studies 1332 patients	37 (12, 63) I^2^ = 98.3% 5 studies 663 patients
Sensitivity analyses									
Excluding studies with less sample representativeness	24 (6, 42) I^2^ = 98.2% 3 studies 2303 patients	3 (2, 5) I^2^ = 0% 2 studies 476 patients	29 (15, 44) I^2^ = 82.7% 3 studies 220 patients	-	36 (27, 45) I^2^ = 72.4% 3 studies 468 patients	-	-	31 (8, 55) I^2^ = 90.1% 2 studies 160 patients	-
Excluding studies with less comparable respondent and non-respondent comparability	-	-	45 (37, 54) I^2^ = 68.1% 3 studies 482 patients	44 (29, 59) I^2^ = 91.9% 3 studies 996 patients	-	-	-	42 (17, 66) I^2^ = 96.9% 3 studies 482 patients	-
Excluding studies only using female sample	16 (11, 21) I^2^ = 79.8% 6 studies 2383 patients	16 (4, 28) I^2^ = 95.0% 4 studies 496 patients	27 (17, 36) I^2^ = 92.9% 9 studies 1195 patients	44 (35, 54) I^2^ = 85.6% 4 studies 1168 patients	39 (29, 49) I^2^ = 88.2% 6 studies 781 patients	34 (2, 65) I^2^ = 98.8% 3 studies 545 patients	5 (−2, 12) I^2^ = 85.9% 2 studies 266 patients	33 (27, 39) I^2^ = 79.4% 8 studies 1135 patients	12 (2, 23) I^2^ = 76.5% 2 studies 166 patients
Subgroup analyses									
Sample size									
<200	22 (14, 31) I^2^ = 90.5% 8 studies 807 patients	14 (5, 23) I^2^ = 93.3% 5 studies 596 patients	29 (22, 36) I^2^ = 81.1% 10 studies 878 patients	38 (28, 48) I^2^ = 86.3% 6 studies 1008 patients	39 (25, 52) I^2^ = 90.5% 5 studies 496 patients	41 (−14, 96) I^2^ = 99.2% 2 studies 260 patients	5 (0, 9) I^2^ = 72.1% 3 studies 326 patients	43 (31, 55) I^2^ = 91.8% 8 studies 736 patients	30 (9, 52) I^2^ = 96.0% 4 studies 337 patients
≥200	27 (2, 57) I^2^ = 99.1% 2 studies 2153 patients	-	35 (2, 67) I^2^ = 98.8% 2 studies 596 patients	39 (36, 42) I^2^ = 0.0% 2 studies 632 patients	-	-	-	28 (16, 40) I^2^ = 90.8% 2 studies 596 patients	-
Overall quality									
<3 points (low quality)	23 (13, 34) I^2^ = 91.8% 7 studies 657 patients	18 (6, 29) I^2^ = 93.2% 4 studies 446 patients	26 (18, 33) I^2^ = 77.5% 6 studies 581 patients	34 (28, 40) I^2^ = 45.5% 4 studies 443 patients	42 (21, 63) I^2^ = 93.8% 3 studies 313 patients	41 (−14, 96) I^2^ = 99.2% 2 studies 260 patients	5 (0, 9) I^2^ = 72.1% 3 studies 326 patients	42 (32, 52) I^2^ = 82.5% 5 studies 499 patients	30 (9, 52) I^2^ = 96.0% 4 studies 337 patients
≥3 points (high quality)	26 (6, 42) I^2^ = 98.2% 3 studies 2303 patients	3 (2, 5) I^2^ = 0% 2 studies 476 patients	34 (20, 48) I^2^ = 95.0% 6 studies 893 patients	42 (33, 52) I^2^ = 87.9% 4 studies 1197 patients	36 (27, 45) I^2^ = 72.4% 3 studies 468 patients	-	-	38 (23, 53) I^2^ = 95.5% 5 studies 833 patients	-
Publication year									
1990s	-	-	-	-	-	-	-	-	-
2000s	33 (17, 50) I^2^ = 91.0% 3 studies 457 patients	-	25 (17, 33) I^2^ = 81.3% 5 studies 660 patients	28 (23, 34) I^2^ = 0.0% 2 studies 261 patients	30 (10, 51) I^2^ = 86.8% 2 studies 141 patients	-	-	39 (22, 57) I^2^ = 95.0% 4 studies 600 patients	56 (39, 73) I^2^ = 86.3% 2 studies 397 patients
2010-	21 (14, 29) I^2^ = 91.5% 6 studies 2424 patients	11 (2, 19) I^2^ = 92.0% 4 studies 517 patients	35 (22, 48) I^2^ = 93.1% 6 studies 734 patients	42 (35, 48) I^2^ = 78.6% 6 studies 1379 patients	43 (32, 55) I^2^ = 88.5% 4 studies 640 patients	34 (2, 65) I^2^ = 98.8% 3 studies 545 patients	5 (0, 9) I^2^ = 72.1% 3 studies 326 patients	41 (26, 56) I^2^ = 93.8% 5 studies 652 patients	34 (2, 67) I^2^ = 96.1% 2 studies 187 patients
Country of origin									
North America	22 (8, 37) I^2^ = 97.3% 4 studies 2363 patients	3 (2, 5) I^2^ = 0% 2 studies 476 patients	-	38 (31, 45) I^2^ = 83.9% 7 studies 1439 patients	30 (10, 51) I^2^ = 86.8% 2 studies 141 patients	-	-	-	-
Asia	21 (0, 41) I^2^ = 96.0% 3 studies 259 patients	21 (−3, 44) I^2^ = 93.7% 2 studies 179 patients	26 (18, 34) I^2^ = 85.4% 6 studies 767 patients	-	50 (31, 69) I^2^ = 91.3% 2 studies 385 patients	34 (2, 65) I^2^ = 98.8% 3 studies 545 patients	-	37 (23, 51) I^2^ = 94.4% 6 studies 767 patients	29 (−13, 72) I^2^ = 98.2% 2 studies 179 patients
Europe	-	-	33 (17, 49) I^2^ = 93.8% 4 studies 565 patients	-	-	-	-	44 (28, 61) I^2^ = 91.9% 3 studies 505 patients	-
South America	31 (12, 50) I^2^ = 85.3% 2 studies 158 patients	-	-	-	-	-	-	-	32 (4, 60) I^2^ = 93.5% 2 studies 158 patients

The first line in each set of data is percentage prevalence (95% CI)

*DSM* Diagnostic and Statistical Manual of Mental Disorders, *ICD* International Classification of Diseases, *HADS* Hospital Anxiety and Depression Scale, *CES-D* Centre for Epidemiological Studies Depression Scale, *BDI* Beck Depression Inventory

### Associated study variables

We used Pearson’s and Spearmen’s correlation analyses to assess the association between variables including mean/medium disease duration, proportion of female participants, mean/medium age, representativeness, sample size, comparability, overall quality, country of origin, publication year, and the prevalence of depression and anxiety. Table 5 indicated that more recent publications was significantly associated with increased depression prevalence (*r* = 0.26, *P* = 0.04). No study characteristics presented a significant association with anxiety prevalence estimate.

**Table 5:** Pearson's and Spearmen’s correlation between study characteristics and prevalence estimates

Study characteristic	Depression prevalence estimate			Anxiety prevalence estimate		
	No. of studies	*r*	*P*	No. of studies	*r*	*P*
Female, %	59	0.03	0.84	24	0.07	0.76
Mean/medium age, year	55	−0.13	0.35	23	−0.18	0.94
Mean/medium disease duration, year	53	−0.07	0.64	21	0.24	0.29
Representativeness	59	0.03	0.85	24	0.08	0.70
Sample size	59	0.12	0.38	24	0.01	0.97
Comparability	59	0.24	0.07	24	−0.11	0.61
Overall quality	59	0.13	0.33	24	−0.10	0.64
Country of origin	59	0.01	0.92	24	−0.10	0.63
Publication year	59	0.26^*^	0.04	24	−0.04	0.84

^*^Significant at a *P* <0.05 level

### Assessment of publication bias

Assessment of publication bias indicated significant publication bias, according to the Egger’s test, in studies reporting depression according to HADS with thresholds of 8 and CES-D with a cutoff of 16 or more [Egger: bias = 0.81 (95% CI: 0.04, 1.58), *P* = 0.04, and Egger: bias = 2.79 (95% CI: 0.61, 4.97), *P* = 0.02, respectively]. There was no significant evidence of publication bias in any other analyses (see Additional file 5).

## Discussion

This systematic review and meta-analysis of 59 studies involving 10828 adult SLE patients demonstrated that a few studies using gold standard clinical interviews (DSM and/or ICD) reported that major depression and anxiety were presented in 24% and 37% among SLE patients, respectively. The majority of studies using screening tools found that significant depression were presented in 30% using the HADS a cutoff of 8 or more and 39% using the 21 Item-BDI with thresholds of 14. This study also found that more recent publications was significantly associated with increased depression prevalence among SLE patients. Furthermore, the prevalence of anxiety was 40% according to the HADS with thresholds of 8. These prevalence estimates are significantly higher than those observed in the general population [23, 24] and other rheumatic and connective tissue diseases [15, 25, 26]. Furthermore, these findings demonstrated that SLE patients tended to have a higher prevalence of anxiety than depression, which was in line with previous studies [27, 28]. Such discrepancy could be explained by the differences in time frames when these studies were performed, disease characteristics, social and cultural contexts of the lupus patients and tools used for assessing depression or anxiety. Because the development of depression and/or anxiety could result in increased incidence of cardiovascular diseases [5], decreased quality of life [9, 10], and a higher risk of premature mortality [11] among SLE patients, these findings highlighted an important issue in health education for this population.

Neuropsychiatric (NP) disorders appeared in about 70% of the patients diagnosed with SLE [29]. Previous meta-analyses have assessed the prevalence of the 19 NP syndromes defined by the American College of Rheumatology (ACR) in 1999 among SLE patients [30]. However, there were a wide variety of neurologic and psychiatric manifestations of SLE, which extended beyond those identified in the 1999 ACR classification criteria for SLE [31]. Several attempts have been made to devise a classification of NP-SLE manifestations because there were controversies regarding the inclusion of mood disorders in the 1999 ACR NP-SLE criteria [31, 32]. That’s why we excluded the studies investigating neuropsychiatric syndromes among SLE patients in this meta-analysis.

Although studies varied widely in terms of quality, our sensitivity analyses suggested that depression and/or anxiety prevalence estimates (except dysthymic disorder estimates) were reasonably stable. Variation in study sample size contributed importantly to the observed heterogeneity in the data. Studies with sample size <200 had higher anxiety estimates according to the HADS with thresholds of 8. Furthermore, studies with lower total overall quality scores yielded higher dysthymic disorder estimates according to DSM and/or ICD. Country, publication year, age, and gender also contributed to the heterogeneity between studies.

In this meta-analysis, many methods were used for data extraction and synthesis. The gold standard method was diagnostic interviews using DSM or ICD criteria, which were often time consuming and expensive. Therefore, it was not ideal for examining patients in a busy hospital environment [33]. Alternatively, self-report screening tools might be used, because they were quick and easy to complete and cheaper to use than diagnostic interviews. However, prevalence estimates using screening tools were often overestimated, because such tools tended to prioritize sensitivity over specificity [33]. Furthermore, there have not been validation studies to determine the best cut-point for screening tools in SLE patients, and several cut-off scores on self-report tools were often used in many studies. It indicated that the rheumatologists should always report prevalence at conventional cut-points, and screen for depression and anxiety among SLE patients according to the social and cultural contexts of the rheumatologists and SLE patients in clinical practice.

There are, however, additional important shortcomings in the evidence on prevalence of depression in SLE that need to be addressed. First, a substantial amount of the heterogeneity among the studies remained unexplained by the variables examined. Unexamined factors, such as gender, age, disease duration, might contribute to the risk for depression and/or anxiety symptom among SLE patients. Second, the data were derived from studies that used different designs and involved different groups of patients (e.g., from different countries), which might result in heterogeneity among the studies. Third, we did not look for healthy subjects in each study reporting the prevalence of depression or anxiety in SLE patients, which should be addressed in future research.

## Conclusions

The prevalence of depression and anxiety was high in adult SLE patients. It indicated that rheumatologists should screen for depression and anxiety in their patients, and they should refer them to mental health providers in order to identify effective strategies for preventing and treating depression and anxiety among SLE patients.

## Additional files

Additional file 1: Search Terms. (DOCX 10 kb)
Additional file 2: Summaries of symptom thresholds required for diagnosis of depression/anxiety. (DOCX 19 kb)
Additional file 3: The list of 59 studies included in the meta-analysis. (DOCX 19 kb)
Additional file 4: Quality Assessment. (DOCX 19 kb)
Additional file 5: Assessment of Publication Bias. (DOCX 56 kb)
