# Supplementary material for: Prevalence of depression and anxiety in systemic lupus erythematosus: a systematic review and meta-analysis
Source: BMC Psychiatry. 2017 Feb 14;17:70. doi: 10.1186/s12888-017-1234-1 (PMC5310017; doi:10.1186/s12888-017-1234-1)
Supplement: Additional file 1: — Search Terms. (DOCX 10 kb) [file 12888_2017_1234_MOESM1_ESM.docx]

**Additional file 1**: Search Terms

Embase/PsyInfo Web of Science/PubMed

(anxiety or anxiety disorder$ or depress* or depress* disorder$ or affective disorder$ or mood disorder$ or adjustment disorder$ or affective symptom$ or dysthymi*) AND (systemic lupus erythematosus or lupus or SLE)
