# Supplementary material for: Prevalence of depression and anxiety in systemic lupus erythematosus: a systematic review and meta-analysis
Source: BMC Psychiatry. 2017 Feb 14;17:70. doi: 10.1186/s12888-017-1234-1 (PMC5310017; doi:10.1186/s12888-017-1234-1)
Supplement: Additional file 2: — Summaries of symptom thresholds required for diagnosis of depression/anxiety. (DOCX 19 kb) [file 12888_2017_1234_MOESM2_ESM.docx]

| **Additional file 2**: Summaries of symptom thresholds required for diagnosis of depression/anxiety | | |
| --- | --- | --- |
| **Measures of Mood/Anxiety Disorders** | **Mood/Anxiety Disorder** | **Description** |
| ICD-9/10 | Major Depression | For a minimum of two weeks, patient must exhibit at least two of the following criteria: depressed mood; loss of interest in daily activities; decreased energy or increased fatigability. The patient must also show signs of at least two of the remaining seven symptoms: loss of confidence & self-esteem; self-reproach or feelings of guilt; recurrent thoughts of death or suicide; diminished ability to concentrate; change in psychomotor activity; sleep disturbance of any type; change in appetite. |
|  | Minor Depression | For a patient to be diagnosed with minor depression, they must exhibit at least two of the following symptoms: lowered mood; decrease in activity participation; reduced interest or enjoyment in activities; impaired concentration; fatigue; sleep disruption; changed appetite; reduced self-esteem/confidence; feelings of worthlessness; psychomotor agitation/retardation; loss of libido. The patient must show signs of depression, but be able to continue with daily life without significant interference. |
|  | Dysthymic Disorder | A chronic depressed mood lasting several years, but not severe enough to be diagnosed as depression. |
|  | Adjustment Disorder | A subjective experience of distress or emotional disturbance which interferes with social functions, arising in a period of adaptation to stressor(s). The stressor(s) may have affected the integrity of social network or values or have represented a developmental transition or crisis. Symptoms include depressed mood, anxiety, inability to cope or plan ahead, and disability in performance of normal routine. |
| DSM-III | Major Depression | For a minimum of 2 weeks, patient must exhibit at least 1 of the following symptoms: depressed mood; reduced interest in daily activities. Additionally, the patient must experience at least 4 of the following symptoms: change in weight/appetite; insomnia/hypersomnia; psychomotor agitation or retardation; fatigue; loss of interest in usual activities, or decrease in sexual drive; feelings of guilt/worthlessness; reduced concentration; suicidal ideation. These symptoms must represent a change from normal behaviour, must not be superimposed on another psychiatric disorder, nor due to bereavement. |
|  | Dysthymic Disorder | For a minimum of 2 weeks, the individual has suffered from symptoms of depressive syndrome, but that are not of sufficient severity or duration to meet criteria for major depression. The manifestations of depressive syndrome may be persistent, or separated by periods of normal mood, lasting up to a few weeks at a time. During depressive periods, there is either prominent depressed mood or loss of interest in daily activities. During depressive periods, at least 3 of the following symptoms are present: insomnia/hypersomnia; fatigue; low self-esteem; decreased effectiveness at work/school; reduced attention/concentration; social withdrawal; loss of interest/ enjoyment in activities; inability to respond with pleasure to praise; less/more active or talkative than usual; pessimistic attitude towards the future; tearfulness; suicidal thoughts. |
| DSM-IV | Major Depression | For a minimum of 2 weeks, patient must exhibit at least 1 of the following symptoms: depressed mood; reduced interest in daily activities. Additionally, patient must have at least 5 of the following symptoms: depressed mood; diminished pleasure in daily activities; weight/appetite change; energy loss/fatigue; insomnia/hypersomnia; psychomotor agitation/retardation; feelings of guilt/worthlessness; reduced concentration; suicidal ideation. These symptoms must represent a change from normal behaviour, must not be superimposed on another psychiatric disorder, nor due to bereavement. These symptoms must also not meet the criteria for a Mixed Depressive Episode. |
|  | Dysthymic Disorder | Depressed mood for most of the day, most days, indicated by subjective account or noticed by others for at least 2 years. While depressed, at least two of the following symptoms must be experienced: poor appetite/overeating; insomnia/hypersomnia; fatigue; low self-esteem; reduced concentration or indecisiveness; feelings of hopelessness. During the 2 year period of the disturbance, the person has never been without symptoms for more than 2 months at a time. No Major Depressive episode has been present during the first 2 years of disturbance. There has never been a manic episode, mixed episode, or hypomanic episode, and criteria have never been met for cyclothymic disorder. The disturbance does not occur during the course of a chronic Psychotic disorder and symptoms are not due to the direct physiological effects of a substance or medical condition. The symptoms cause clinically significant distress or impairment in social, occupational or other important areas of functioning. |
|  | Adjustment Disorder | The development of emotional or behavioural symptoms in response to an identifiable stressor(s) occurring within 3 months of the onset of the stressor(s). These symptoms or behaviours are clinically significant as evidenced by the following: marked distress that is in excess of what would be expected from exposure to the stressor; significant impairment in social or occupational functioning. The disturbance does not meet the criteria for another mental disorder, is not an exacerbation of a pre-existing disorder, nor do the symptoms represent bereavement. Once the stressor has terminated, the symptoms do not persist for more than 6 months. |
| Beck Depression Inventory (BDI) | BDI | Well-validated 21-item measure designed to assess depressive symptomatology. Scores for the items range between 0-3, reflecting the frequency with which the problem occurs. A total score is obtained by summing the scores for the 21 questions (range = 0-63). A score of 14 is usually accepted as indicative of moderate depressive symptoms, and 25 or greater is associated with clinically significant major depression. |
| Beck Anxiety Inventory (BAI) | BAI | Well-validated 21-item measure designed to assess anxiety symptomatology. Scores for the items range between 0-3, reflecting the frequency with which the problem occurs. A total score is obtained by summing the scores for the 21 questions (range = 0-63). A score of 14 is usually accepted as indicative of moderate anxiety symptoms, and 25 or greater is associated with clinically significant anxiety. |
| Center of Epidemiological Studies Depression Scale (CES-D) | CES-D | Consists of 20 items that categorize the intensity of depressive symptoms in the following spheres: depressed mood; decreased pleasure in everyday activities; loss of appetite; sleep disturbance; agitation/psychomotor delay; fatigue; guilt/worry; though disorder; suicidal ideation. Each item is given a value of 0 ("never") to 4 ("daily") depending on its presence in the preceding 2 weeks. A global score of 0-60 is computed and a cut-off value of 16 is usually required to indicate possible depressive symptoms. The CES-D classifies patients into 5 grades of probability of the presence of major depression: 1. asymptomatic patient; 2. subthreshold depressive disorder; 3. possible depressive disorder; 4. probable depressive disorder; 5. clinically significant symptoms of episodes of major depression. An abbreviated version of the original scale, using 10-items, is also frequently used, with the shorter version having close agreement with the original. |
| Hospital Anxiety and Depression Scale (HADS) | HADS | Constructed to assess anxiety and depression in patients with somatic diseases. It consists of 14 short statements. The patient indicates how much is applies to their situation during the last week, "not at all", "sometimes", "often", or "very often". Seven statements measure anxiety and 7 depression, independent of each other. Normal is usually defined by scores of 0-7, borderline abnormal ranges from 8-10 and abnormal usually ranges 11-21. |
| Patient Health Questionnaire (PHQ-2) | PHQ-2 | The PHQ-2 includes questions about the frequency of depressed mood and anhedonia over the previous 2 weeks and consists of 2 questions from the Patient Health Questionnaire-9. Each question is rated on a scale from 0 (not at all) to 3 (nearly every day). The PHQ-2 overall score ranges from 0 to 6. The intention of the PHQ-2 is not to establish a diagnosis, but to screen for depression. A PHQ-2 score ≥3 has a sensitivity of 83% and a specificity of 92% for detecting major depression. |
| Patient Health Questionnaire (PHQ-9) | PHQ-9 | The PHQ-9 comprises 9 items, which correspond to the 9 diagnostic criteria of major depressive disorder. Patients are asked to what extent they have experienced the symptoms within the past 2 weeks, with the possible answers: "not at all", "several days", "more than half the days", or "nearly every day". Commonly used cut-off scores are 5-9 (mild depressive symptoms), 10-14 (moderate depressive symptoms), 15-19 (moderate-severe depressive symptoms) and >20 (severe depressive symptoms). |
| Zung Self-Rating Depression Scale (SDS) | SDS | Consists of 20 questions, with patients asked to mark one of four answers ("never", "sometimes", "frequently", and "always") for each question. The items are scored from 1-4, creating a total score ranging between 20 and 80. The cut-off point usually required to indicate depressive symptoms is 44. |
| Zung Self-Rating Anxiety Scale (SAS) | SAS | Consists of 20 questions, with patients asked to mark one of four answers ("never", "sometimes", "frequently", and "always") for each question. The items are scored from 1-4, creating a total score ranging between 20 and 80. The cut-off point usually required to indicate anxiety symptoms is 44. |
| Symptoms Checklist-90-Revised (SCL-90-R) | SCL-90-R | It consists of 90 questions and is a self-reported measurement of  current psychological symptom status used primarily with psychiatric and medical patients. Originally, the SCL-90-R was devised for psychiatric screening of general populations, and it was found by Duckro and his coworkers to be a useful psychological scale for use with chronic pain patients. |
| Hamilton Depression Scale (HDS) | HDS | It is a multiple item questionnaire used to provide an indication of depression, and as a guide to evaluate recovery. The questionnaire is designed for adults and is used to rate the severity of their depression by probing mood, feelings of guilt, suicide ideation, insomnia, agitation or retardation, anxiety, weight loss, and somatic symptoms. The original 1960 version contains 17 items to be rated, but four other questions are not added to the total score and are used to provide additional clinical information. Each item on the questionnaire is scored on a 3 or 5 point scale, depending on the item, and the total score is compared to the corresponding descriptor. |
| Hamilton Anxiety Scale (HAS) | HAS | It is a psychological questionnaire used by clinicians to rate the severity of a patient's anxiety. Anxiety can refer to things such as “a mental state…a drive…a response to a particular situation…a personality trait…and a psychiatric disorder.” The scale consists of 14 items designed to assess the severity of a patient’s anxiety. Each of the 14 items contains a number of symptoms, and each group of symptoms is rated on a scale of zero to four, with four being the most severe. All of these scores are used to compute an overarching score that indicates a person’s anxiety severity. |
| Cattell questionnaire |  | This questionnaire consists of 40 questions each scored between  0 and 2, the higher score marks more severe anxiety. Scores between 21 and 40 suggest symptomatic anxiety and scores between 1 and 21 suggest latent anxiety. |
