# Supplementary material for: Prevalence of depression and anxiety in systemic lupus erythematosus: a systematic review and meta-analysis
Source: BMC Psychiatry. 2017 Feb 14;17:70. doi: 10.1186/s12888-017-1234-1 (PMC5310017; doi:10.1186/s12888-017-1234-1)
Supplement: Additional file 3: — The list of 59 studies included in the meta-analysis. (DOCX 19 kb) [file 12888_2017_1234_MOESM3_ESM.docx]

**Additional file 3:** The list of 59 studies included in the meta-analysis

**Abdul-Sattar 2015**

Abdul-Sattar AB, Abou El Magd SA. Determinants of medication non-adherence in Egyptian patients with systemic lupus erythematosus: Sharkia Governorate. Rheumatology international. 2015;35(6):1045-51.

**Appenzeller 2009**

Appenzeller S, Cendes F, Costallat LT. Cognitive impairment and employment status in systemic lupus erythematosus: a prospective longitudinal study. Arthritis and rheumatism. 2009;61(5):680-7.

**Bachen 2009**

Bachen EA, Chesney MA, Criswell LA. Prevalence of mood and anxiety disorders in women with systemic lupus erythematosus. Arthritis and rheumatism. 2009;61(6):822-9.

**Bogdanovic 2015**

Bogdanovic G, Stojanovich L, Djokovic A, Stanisavljevic N. Physical Activity Program Is Helpful for Improving Quality of Life in Patients with Systemic Lupus Erythematosus. Tohoku Journal Of Experimental Medicine. 2015;237(3):193-9.

**Calderon 2014**

Calderon J, Flores P, Babul M, Aguirre JM, Slachevsky A, Padilla O, et al. Systemic lupus erythematosus impairs memory cognitive tests not affected by depression. Lupus. 2014;23(10):1042-53.

**Chin 1993**

Chin CN, Cheong I, Kong N. Psychiatric disorder in Malaysians with systemic lupus erythematosus. Lupus. 1993;2(5):329-32.

**Cho 2014**

Cho JH, Chang SH, Shin NH, Choi BY, Oh HJ, Yoon MJ, et al. Costs of illness and quality of life in patients with systemic lupus erythematosus in South Korea. Lupus. 2014;23(9):949-57.

**Da Costa 2005**

Da Costa D, Bernatsky S, Dritsa M, Clarke AE, Dasgupta K, Keshani A, et al. Determinants of sleep quality in women with systemic lupus erythematosus. Arthritis and rheumatism. 2005;53(2):272-8.

**Doria 2004**

Doria A, Rinaldi S, Ermani M, Salaffi F, Iaccarino L, Ghirardello A, et al. Health-related quality of life in Italian patients with systemic lupus erythematosus. II. Role of clinical, immunological and psychological determinants. Rheumatology. 2004;43(12):1580-6.

**Duvdevany 2011**

Duvdevany I, Cohen M, Minsker-Valtzer A, Lorber M. Psychological correlates of adherence to self-care, disease activity and functioning in persons with systemic lupus erythematosus. Lupus. 2011;20(1):14-22.

**Garcia Carrasco 2013**

Garcia Carrasco M, Mendoza Pinto C, Lopez Colombo A, Mendez Martinez S, Andari Sawaya R, Munoz Guarneros M, et al. Irritable bowel syndrome-type symptoms in female patients with mild systemic lupus erythematosus: frequency, related factors and quality of life. Neurogastroenterology And Motility. 2013;25(12):958-66.

**Garcia-Carrasco 2011**

Garcia-Carrasco M, Mendoza-Pinto C, Riebeling C, Sandoval-Cruz M, Nava A, Etchegaray-Morales I, et al. Influence of prevalent vertebral fractures on the quality of life of patients with systemic lupus erythematosus. The Israel Medical Association journal : IMAJ. 2011;13(6):333-7.

**Greco 2009**

Greco CM, Kao AH, Sattar A, Danchenko N, Maksimowicz-McKinnon KM, Edmundowicz D, et al. Association between depression and coronary artery calcification in women with systemic lupus erythematosus. Rheumatology. 2009;48(5):576-81.

**Hanly 2015**

Hanly JG, Su L, Urowitz MB, Romero-Diaz J, Gordon C, Bae S-C, et al. Mood Disorders in Systemic Lupus Erythematosus: Results From an International Inception Cohort Study. Arthritis & rheumatology. 2015;67(7):1837-47.

**Harrison 2006**

Harrison MJ, Ravdin LD, Lockshin MD. Relationship between serum NR2a antibodies and cognitive dysfunction in systemic lupus erythematosus. Arthritis and rheumatism. 2006;54(8):2515-22.

**Huang 2007**

Huang HC, Chou CT, Lin KC, Chao YF. The relationships between disability level, health-promoting lifestyle, and quality of life in outpatients with systemic lupus erythematosus. The journal of nursing research : JNR. 2007;15(1):21-32.

**Iverson 2002**

Iverson GL. Screening for depression in systemic lupus erythematosus with the British Columbia Major Depression Inventory. Psychological reports. 2002;90(3 Pt 2):1091-6.

**Jarpa 2011**

Jarpa E, Babul M, Calderon J, Gonzalez M, Martinez ME, Bravo-Zehnder M, et al. Common mental disorders and psychological distress in systemic lupus erythematosus are not associated with disease activity. Lupus. 2011;20(1):58-66.

**Julian 2011**

Julian LJ, Gregorich SE, Tonner C, Yazdany J, Trupin L, Criswell LA, et al. Using the Center for Epidemiologic Studies Depression Scale to screen for depression in systemic lupus erythematosus. Arthritis care & research. 2011;63(6):884-90.

**Jung 2015**

Jung J-Y, Nam J-Y, Kim H-A, Suh C-H. Elevated Salivary Alpha-Amylase Level, Association Between Depression and Disease Activity, and Stress as a Predictor of Disease Flare in Systemic Lupus Erythematosus A Prospective Case-Control Study. Medicine. 2015;94(30).

**Karimifar 2013**

Karimifar M, Sharifi I, Shafiey K. Anti-ribosomal P antibodies related to depression in early clinical course of systemic lupus erythematosus. Journal of research in medical sciences : the official journal of Isfahan University of Medical Sciences. 2013;18(10):860-4.

**Karol 2013**

Karol DE, Criscione-Schreiber LG, Lin M, Clowse ME. Depressive symptoms and associated factors in systemic lupus erythematosus. Psychosomatics. 2013;54(5):443-50.

**Katz 2011**

Katz P, Yazdany J, Julian L, Trupin L, Margaretten M, Yelin E, et al. Impact of obesity on functioning among women with systemic lupus erythematosus. Arthritis care & research. 2011;63(10):1357-64.

**Kheirandish 2015**

Kheirandish M, Faezi ST, Paragomi P, Akhlaghi M, Gharibdoost F, Shahali A, et al. Prevalence and severity of depression and anxiety in patients with systemic lupus erythematosus: An epidemiologic study in Iranian patients. Modern rheumatology / the Japan Rheumatism Association. 2015;25(3):405-9.

**Kim 2015**

Kim SS, Mancuso CA, Huang WT, Erkan D. Social capital: a novel platform for understanding social determinants of health in systemic lupus erythematosus. Lupus. 2015;24(2):122-9.

**Kotsis 2014**

Kotsis K, Voulgari PV, Tsifetaki N, Drosos AA, Carvalho AF, Hyphantis T. Illness perceptions and psychological distress associated with physical health-related quality of life in primary Sjogren's syndrome compared to systemic lupus erythematosus and rheumatoid arthritis. Rheumatology international. 2014;34(12):1671-81.

**Lapteva 2006**

Lapteva L, Nowak M, Yarboro CH, Takada K, Roebuck-Spencer T, Weickert T, et al. Anti-N-methyl-D-aspartate receptor antibodies, cognitive dysfunction, and depression in systemic lupus erythematosus. Arthritis and rheumatism. 2006;54(8):2505-14.

**Lisitsyna 2014**

Lisitsyna T, Veltishchev D, Seravina O, Kovalevskaya O, Marchenko A, Nasonov E. Psychiatric morbidity in systemic lupus erythematosus patients. Clinical and experimental rheumatology. 2014;32(4):S45-S6.

**Mak 2011**

Mak A, Tang CS, Chan MF, Cheak AA, Ho RC. Damage accrual, cumulative glucocorticoid dose and depression predict anxiety in patients with systemic lupus erythematosus. Clinical rheumatology. 2011;30(6):795-803.

**Maneeton 2013**

Maneeton B, Maneeton N, Louthrenoo W. Prevalence and predictors of depression in patients with systemic lupus erythematosus: a cross-sectional study. Neuropsychiatric disease and treatment. 2013;9:799-804.

**Mirbagher 2016**

Mirbagher L, Gholamrezaei A, Hosseini N, Bonakdar ZS. Sleep quality in women with systemic lupus erythematosus: contributing factors and effects on health-related quality of life. International journal of rheumatic diseases. 2016;19(3):305-11.

**Monaghan 2007**

Monaghan SM, Sharpe L, Denton F, Levy J, Schrieber L, Sensky T. Relationship between appearance and psychological distress in rheumatic diseases. Arthritis and rheumatism. 2007;57(2):303-9.

**Montero-Lopez 2016**

Montero-Lopez E, Santos-Ruiz A, Navarrete-Navarrete N, Ortego-Centeno N, Perez-Garcia M, Peralta-Ramirez MI. The effects of corticosteroids on cognitive flexibility and decision-making in women with lupus. Lupus. 2016.

**Nery 2008**

Nery FG, Borba EF, Viana VST, Hatch JP, Soares JC, Bonfa E, et al. Prevalence of depressive and anxiety disorders in systemic lupus erythematosus and their association with anti-ribosomal P antibodies. Progress in neuro-psychopharmacology & biological psychiatry. 2008;32(3):695-700.

**Neville 2014**

Neville C, DaCosta D. Toward the development of a lupus interactive navigator to facilitate patients and their health care providers in the management of lupus: results of web-based surveys. 2014;3(4):e65.

**Palagini 2014**

Palagini L, Tani C, Bruno RM, Gemignani A, Mauri M, Bombardieri S, et al. Poor sleep quality in systemic lupus erythematosus: does it depend on depressive symptoms? Lupus. 2014;23(13):1350-7.

**Panopalis 2010**

Panopalis P, Gillis JZ, Yazdany J, Trupin L, Hersh A, Julian L, et al. Frequent use of the emergency department among persons with systemic lupus erythematosus. Arthritis care & research. 2010;62(3):401-8.

**Pettersson 2015**

Pettersson S, Bostroem C, Eriksson K, Svenungsson E, Gunnarsson I, Henriksson EW. Lifestyle habits and fatigue among people with systemic lupus erythematosus and matched population controls. Lupus. 2015;24(9):955-65.

**Postal 2016**

Postal M, Lapa AT, Sinicato NA, de Oliveira Pelicari K, Peres FA, Costallat LT, et al. Depressive symptoms are associated with tumor necrosis factor alpha in systemic lupus erythematosus. Journal of neuroinflammation. 2016;13:5.

**Radhakrishan 2011**

Radhakrishan R, Galgalirb, Shobhav. Prevalence of psychiatric morbidity among patients with systemic lupus erythematosus. Indian Journal of Psychiatry. 2011;53(5):S45-S6.

**Roebuck-Spencer 2006**

Roebuck-Spencer TM, Yarboro C, Nowak M, Takada K, Jacobs G, Lapteva L, et al. Use of computerized assessment to predict neuropsychological functioning and emotional distress in patients with systemic lupus erythematosus. Arthritis and rheumatism. 2006;55(3):434-41.

**Segal 2012**

Segal BM, Thomas W, Zhu X, Diebes A, McElvain G, Baechler E, et al. Oxidative stress and fatigue in systemic lupus erythematosus. Lupus. 2012;21(9):984-92.

**Sehlo 2013**

Sehlo MG, Bahlas SM. Perceived illness stigma is associated with depression in female patients with systemic lupus erythematosus. Journal of psychosomatic research. 2013;74(3):248-51.

**Sfikakis 1998**

Sfikakis PP, Mitsikostas DD, Manoussakis MN, Foukaneli D, Moutsopoulos HM. Headache in systemic lupus erythematosus: a controlled study. British journal of rheumatology. 1998;37(3):300-3.

**Shakeri 2015**

Shakeri H, Arman F, Hossieni M, Omrani HR, Vahdani A, Shakeri J. Depression, Anxiety and Disease-Related Variables and Quality of Life Among Individuals With Systemic Lupus Erythematosus Living in Kermanshah Province, Iran. Iranian Red Crescent medical journal. 2015;17(12):e31047.

**Shen 2015**

Shen B, He Y, Chen H, Zhao C, Zhu L, Gao Y, et al. Body Image Disturbances Have Impact on the Sexual Problems in Chinese Systemic Lupus Erythematosus Patients. Journal of immunology research. 2015;2015:204513.

**Shortall 1995**

Shortall E, Isenberg D, Newman SP. Factors associated with mood and mood disorders in SLE. Lupus. 1995;4(4):272-9.

**Skare 2014**

Skare T, da Silva Magalhaes VD, Siqueira RE. Systemic lupus erythematosus activity and depression. Rheumatology international. 2014;34(3):445-6.

**Stoll 2001**

Stoll T, Kauer Y, Buchi S, Klaghofer R, Sensky T, Villiger PM. Prediction of depression in systemic lupus erythematosus patients using SF-36 Mental Health scores. Rheumatology. 2001;40(6):695-8.

**Tam 2008**

Tam LS, Wong A, Mok VC, Zhu YE, Kwok LW, Li TK, et al. The relationship between neuropsychiatric, clinical, and laboratory variables and quality of life of Chinese patients with systemic lupus erythematosus. The Journal of rheumatology. 2008;35(6):1038-45.

**Tay 2015**

Tay SH, Cheung PP, Mak A. Active disease is independently associated with more severe anxiety rather than depressive symptoms in patients with systemic lupus erythematosus. Lupus. 2015;24(13):1392-9.

**Tench 2000**

Tench CM, McCurdie I, White PD, D'Cruz DP. The prevalence and associations of fatigue in systemic lupus erythematosus. Rheumatology. 2000;39(11):1249-54.

**Tjensvoll 2011**

Tjensvoll AB, Harboe E, Goransson LG, Beyer MK, Greve OJ, Herigstad A, et al. Migraine is frequent in patients with systemic lupus erythematosus: a case-control study. Cephalalgia : an international journal of headache. 2011;31(4):401-8.

**Utset 2015**

Utset TO, Baskaran A, Segal BM, Trupin L, Ogale S, Herberich E, et al. Work disability, lost productivity and associated risk factors in patients diagnosed with systemic lupus erythematosus. Lupus science & medicine. 2015 2015;2(1):e000058-e.

**van Exel 2013**

van Exel E, Jacobs J, Korswagen LA, Voskuyl AE, Stek M, Dekker J, et al. Depression in systemic lupus erythematosus, dependent on or independent of severity of disease. Lupus. 2013;22(14):1462-9.

**Vina 2015**

Vina ER, Hausmann LR, Utset TO, Masi CM, Liang KP, Kwoh CK. Perceptions of racism in healthcare among patients with systemic lupus erythematosus: a cross-sectional study. Lupus science & medicine. 2015;2(1):e000110.

**Weder-Cisneros 2004**

Weder-Cisneros ND, Tellez-Zenteno JF, Cardiel MH, Guibert-Toledano M, Cabiedes J, Velasquez-Paz AL, et al. Prevalence and factors associated with headache in patients with systemic lupus erythematosus. Cephalalgia : an international journal of headache. 2004;24(12):1031-44.

**Xie 2012**

Xie L-F, Chen P-L, Pan H-F, Tao J-H, Li X-P, Zhang Y-J, et al. Prevalence and correlates of suicidal ideation in SLE inpatients: Chinese experience. Rheumatology international. 2012;32(9):2707-14.

**Zakeri 2012**

Zakeri Z, Shakiba M, Narouie B, Mladkova N, Ghasemi-Rad M, Khosravi A. Prevalence of depression and depressive symptoms in patients with systemic lupus erythematosus: Iranian experience. Rheumatology international. 2012;32(5):1179-87.
