# Supplementary material for: Prevalence of depression and anxiety in systemic lupus erythematosus: a systematic review and meta-analysis
Source: BMC Psychiatry. 2017 Feb 14;17:70. doi: 10.1186/s12888-017-1234-1 (PMC5310017; doi:10.1186/s12888-017-1234-1)
Supplement: Additional file 4: — Quality Assessment. (DOCX 19 kb) [file 12888_2017_1234_MOESM4_ESM.docx]

**Additional file 4**: Quality Assessment

Modified Newcastle-Ottawa scoring guide.

**(1) Representativeness of the sample:**

1 point: Population contained a mixture of specialties at multiple sites.

0 points: Population contained a single specialty at a single site.

**(2) Sample size:**

1 point: Sample size was greater than 200 participants.

0 points: Sample size was less than 200 participants or a convenience sample.

**(3) Non-respondents:**

1 point: Comparability between respondent and non-respondent characteristics was established, and the response rate was satisfactory.

0 points: The response rate was unsatisfactory, the comparability between respondents and non-respondents was unsatisfactory, or there was no description of the response rate or the characteristics of the responders and the non-responders.

**(4) Ascertainment of depression:**

1 point: Validated measurement tool using a validated cutoff score or clinical interview.

0 points: Non-validated measurement tool, or validated measurement tool with non-valid cutoff score, or 2-item PRIME-MD (scored as such due to its low specificity).

**(5) Quality of descriptive statistics reporting:**

1 point: Reported descriptive statistics to describe the population (*e.g.*, age, sex) with proper measures of dispersion (*e.g.*, standard deviation, standard error, range).

0 points: Descriptive statistics were not reported, were incomplete, or did not include proper measures of dispersion.

**Legend:** This scale, the scoring of which ranges from 0 to 5, assesses quality in several domains: sample representativeness and size, comparability between respondents and non-respondents, ascertainment of depressive symptoms, and statistical quality. Studies were judged to be of low risk of bias (≥3 points) or high risk of bias (<3 points).

**Total = /5**

| **Results of Newcastle-Ottawa Risk of Bias Assessment.** | | | | | | |
| --- | --- | --- | --- | --- | --- | --- |
| Study ID | Representativeness | Size | Comparability | Outcome | Statistics | Total |
| Abdul-Sattar 2015 | 0 | 0 | 0 | 1 | 1 | 2 |
| Appenzeller 2009 | 0 | 0 | 0 | 1 | 1 | 2 |
| Bachen 2009 | 1 | 1 | 1 | 1 | 1 | 5 |
| Bogdanovic 2015 | 0 | 0 | 0 | 1 | 1 | 2 |
| Calderon 2014 | 0 | 0 | 0 | 1 | 1 | 2 |
| Cho 2014 | 0 | 1 | 0 | 1 | 1 | 3 |
| Chin 1993 | 0 | 0 | 0 | 1 | 1 | 2 |
| Da Costa 2005 | 0 | 0 | 1 | 1 | 1 | 3 |
| Doria 2004 | 0 | 0 | 0 | 1 | 1 | 2 |
| Duvdevany 2011 | 1 | 0 | 1 | 1 | 1 | 4 |
| García Carrasco 2011 | 0 | 0 | 0 | 1 | 1 | 2 |
| García Carrasco 2013 | 0 | 0 | 0 | 1 | 1 | 2 |
| Greco 2009 | 0 | 0 | 0 | 1 | 1 | 2 |
| Hanly 2015 | 1 | 1 | 0 | 1 | 1 | 4 |
| Harrison 2006 | 0 | 0 | 0 | 1 | 1 | 2 |
| Huang 2007 | 0 | 0 | 0 | 1 | 1 | 2 |
| Iverson 2002 | 0 | 0 | 0 | 1 | 0 | 1 |
| Jarpa 2011 | 0 | 0 | 0 | 1 | 1 | 2 |
| Julian 2011 | 1 | 0 | 0 | 1 | 1 | 3 |
| Jung 2015 | 0 | 0 | 0 | 1 | 1 | 2 |
| Katz 2011 | 0 | 1 | 0 | 1 | 1 | 3 |
| Karol 2013 | 0 | 0 | 0 | 1 | 1 | 2 |
| Karimifar 2013 | 0 | 0 | 0 | 1 | 1 | 2 |
| Kheirandish 2015 | 0 | 0 | 0 | 1 | 1 | 2 |
| Kotsis 2014 | 0 | 0 | 0 | 1 | 1 | 2 |
| Kim 2015 | 0 | 0 | 1 | 1 | 1 | 3 |
| Lapteva 2006 | 0 | 0 | 0 | 1 | 1 | 2 |
| Lisitsyna 2014 | 0 | 0 | 0 | 1 | 1 | 2 |
| Mak 2011 | 0 | 0 | 0 | 1 | 1 | 2 |
| Maneeton 2013 | 0 | 0 | 0 | 1 | 1 | 2 |
| Mirbagher 2016 | 0 | 0 | 1 | 1 | 1 | 3 |
| Monaghan 2007 | 1 | 0 | 0 | 1 | 1 | 3 |
| Montero-Lo´pez 2016 | 0 | 0 | 0 | 1 | 1 | 2 |
| Nery 2008 | 0 | 0 | 0 | 1 | 1 | 2 |
| Neville 2014 | 1 | 1 | 0 | 1 | 1 | 4 |
| Palagini 2014 | 0 | 0 | 1 | 1 | 1 | 3 |
| Panopalis 2010 | 1 | 1 | 1 | 1 | 1 | 5 |
| Pettersson 2015 | 0 | 1 | 1 | 1 | 1 | 4 |
| Postal 2016 | 0 | 0 | 0 | 1 | 1 | 2 |
| Radhakrishan 2011 | 0 | 0 | 0 | 1 | 1 | 2 |
| Roebuck-Spencer 2006 | 0 | 0 | 0 | 1 | 1 | 2 |
| Segal 2012 | 0 | 0 | 0 | 1 | 1 | 2 |
| Sehlo 2013 | 0 | 0 | 0 | 1 | 1 | 2 |
| Sfikakis 1998 | 0 | 0 | 0 | 1 | 1 | 2 |
| Shakeri 2015 | 0 | 0 | 0 | 1 | 1 | 2 |
| Shen 2015 | 0 | 0 | 1 | 1 | 1 | 3 |
| Skare 2014 | 0 | 0 | 0 | 1 | 1 | 2 |
| Shorta1l 1995 | 0 | 0 | 0 | 1 | 1 | 2 |
| Stoll 2001 | 1 | 0 | 0 | 1 | 1 | 3 |
| Tam 2008 | 0 | 1 | 0 | 1 | 1 | 3 |
| Tay 2015 | 0 | 0 | 0 | 1 | 1 | 2 |
| Tench 2000 | 0 | 0 | 0 | 1 | 1 | 2 |
| Tjensvoll 2010 | 0 | 0 | 0 | 1 | 1 | 2 |
| Utset 2014 | 1 | 1 | 0 | 1 | 1 | 4 |
| van Exel 2013 | 1 | 0 | 0 | 1 | 1 | 3 |
| Vina 2015 | 1 | 1 | 0 | 1 | 1 | 4 |
| Weder-Cisneros 2004 | 1 | 0 | 0 | 1 | 1 | 3 |
| Xie 2012 | 1 | 1 | 0 | 1 | 1 | 4 |
| Zakeri 2012 | 0 | 0 | 0 | 1 | 1 | 2 |
