# Supplementary material for: Prevalence of depression and anxiety in systemic lupus erythematosus: a systematic review and meta-analysis
Source: BMC Psychiatry. 2017 Feb 14;17:70. doi: 10.1186/s12888-017-1234-1 (PMC5310017; doi:10.1186/s12888-017-1234-1)
Supplement: Additional file 5: — Assessment of Publication Bias. (DOCX 56 kb) [file 12888_2017_1234_MOESM5_ESM.docx]

**Additional file 5**: Assessment of Publication Bias

Major Depression (DSM and/or ICD)


Egger: bias = 3.88 (95% CI: -0.67, 8.43), *P* = 0.09

Dysthymic Disorder (DSM and/or ICD)

Egger: bias = -0.51 (95% CI: -1.56, 0.54), *P* = 0.25

HADS (≥8)-depression

Egger: bias = 0.81 (95% CI: 0.04, 1.58), *P* = 0.04

CES-D (≥16)

Egger: bias = 2.79 (95% CI: 0.61, 4.97), *P* = 0.02

21 Item-BDI (≥14)

Egger: bias = 0.78 (95% CI: -1.13, 2.69), *P* = 0.32

21 Item-BDI (≥21)

Egger: bias = -0.54 (95% CI: -10.16, 9.09), *P* = 0.61

HADS (≥8)-anxiety

Egger: bias = 0.47 (95% CI: -1.34, 2.74), *P* = 0.57

Anxiety Disorder (DSM and/or ICD)

Egger: bias = 0.92 (95% CI: -1.18, 3.01), *P* = 0.26
